# New low-flux mixed matrix membranes that offer superior removal of protein-bound toxins from human plasma

**DOI:** 10.1038/srep34429

**Published:** 2016-10-05

**Authors:** Denys Pavlenko, Esmée van Geffen, Mies J. van Steenbergen, Griet Glorieux, Raymond Vanholder, Karin G. F. Gerritsen, Dimitrios Stamatialis

**Affiliations:** 1 Department of Biomaterials Science and Technology, MIRA Institute for Biomedical Engineering and Technical Medicine, University of Twente, P.O. Box 217, 7500 AE Enschede, The Netherlands; 2 Department of Nephrology and Hypertension, University Medical Centre Utrecht, P.O. Box 85500, 3508 GA Utrecht, The Netherlands; 3 Department of Pharmaceutics, Utrecht Institute for Pharmaceutical Sciences, Utrecht University, P.O. Box 80082, 3508 TB Utrecht, The Netherlands; 4 Ghent University Hospital, Department of Internal Medicine, Nephrology Division, 9000 Ghent, Belgium

## Abstract

Hemodialysis is a widely available and well-established treatment for patients with End Stage Renal Disease (ESRD). However, although life-sustaining, patient mortality rates are very high. Several recent studies corroborated the link between dialysis patients’ outcomes and elevated levels of protein-bound uremic toxins (PBUT) that are poorly removed by conventional hemodialysis. Therefore, new treatments are needed to improve their removal. Recently, our group showed that the combination of dialysis and adsorption on one membrane, the mixed matrix membrane (MMM), can effectively remove those toxins from human plasma. However, these first MMMs were rather large in diameter and their mass transport characteristics needed improvement before application in the clinical setting. Therefore, in this study we developed a new generation of MMMs that have a smaller diameter and optimized characteristics offering superior ability in removing the PBUT indoxyl sulfate (IS) and p-cresyl sulfate (pCS) in comparison to first generation MMMs (30 and 125% respectively), as well as, a commercial dialysis membrane (more than 100% better removal).

Indoxyl sulfate (IS) and p-cresyl sulfate (pCS) are protein-bound uremic toxins (PBUT) that are known to accumulate in end stage kidney disease patients due to their poor removal by conventional hemodialysis^1^. Elevated serum concentrations of PBUTs have been directly associated with vascular disease, progression of kidney disease and high mortality rates in kidney patients^2^,^3^,^4^,^5^,^6^,^7^,^8^,^9^,^10^,^11^,^12^. The main reason for their poor removal is the fact that in dialysis patients 97% of IS and 95% of pCS is bound to albumin^13^, which is retained by the dialysis membrane. Consequently, only the small unbound fraction can pass the membrane. It is clear that to achieve a higher level of removal of these toxins, a modification to existing treatment is urgently needed.

The use of adsorbent technology has always been considered to be a promising alternative to hemodialysis treatment. Although high removal rates have been achieved by adsorption techniques, such as hemoperfusion, for various blood toxins, including PBUTs, these techniques are rarely used in clinical practice^14^, primarily due to limited urea sorption and no control over the fluid balance. However, several recent studies have proposed to improve PBUT removal by combining the hemodialysis and adsorption techniques. For example, Brettschneider *et al*.^15^ showed that fractionated plasma separation and adsorption (FPSA) therapy improves the removal of the IS and pCS by 187 and 127% respectively in comparison to conventional dialyzers. Comparable results for FPSA were also obtained by Meijers and co-workers^16^. In their work, FPSA was found to be superior to high flux hemodialysis in terms of pCS reduction. Alternatively, Sandeman *et al*.^17^ developed a monolith adsorbent device able to reduce the blood concentration of IS and pCS when used complementary to standard hemodialysis. Meyer *et al*.^18^ were also able to markedly improve the *in vitro* removal of p-cresol, p-cresol sulfate and indican from a plasma solution by adding charcoal to the dialysate side of a single-pass dialysis system. A mathematical model suggested that the improvement in PBUT clearance could be attributed to the maintenance of a virtually close to zero concentration of the uremic toxins at the dialysate side of the hemodialysis membranes, thereby maintaining a maximal concentration gradient, the driving force for toxin removal, across the dialyzer during the experiment.

Recently^19^, our group developed and proved the concept of double layer mixed matrix membranes (MMM) that combine dialysis and adsorption in a single step. The MMMs combine the benefits of diffusion and convection, provided by the membrane structure, and adsorption, achieved by activated carbon particles dispersed through the membrane. To avoid blood-sorbent contact, the blood side of the MMM consists of a particle-free polymeric layer. Our first results for removal of small water-soluble toxins and PBUTs by hollow MMM were encouraging^20^. However, these membranes were rather large (internal diameter of around 700 μm) in comparison to the hollow fibers of 200 μm currently used in clinical practice, hampering clinical implementation. Additionally, these membranes had rather large pores, resulting in albumin leakage during convective treatments, which might be considered undesirable.

In this work, we develop a new generation of mixed matrix hollow fiber membranes that are suitable for dialysis treatment. The new membranes are smaller in diameter and do not suffer from albumin leakage, thereby meeting the characteristics of low-flux dialyzers used in the clinic. The performance of the new membranes for removal of creatinine, a small water soluble solute, and of the IS and p-CS is evaluated and compared to first generation of MMM^20^ and to Fresenius F8HPS low flux dialyser membranes, currently used in clinical practice.

## Results and Discussion

### Development of the new hollow fiber membranes

**Figure 1:**
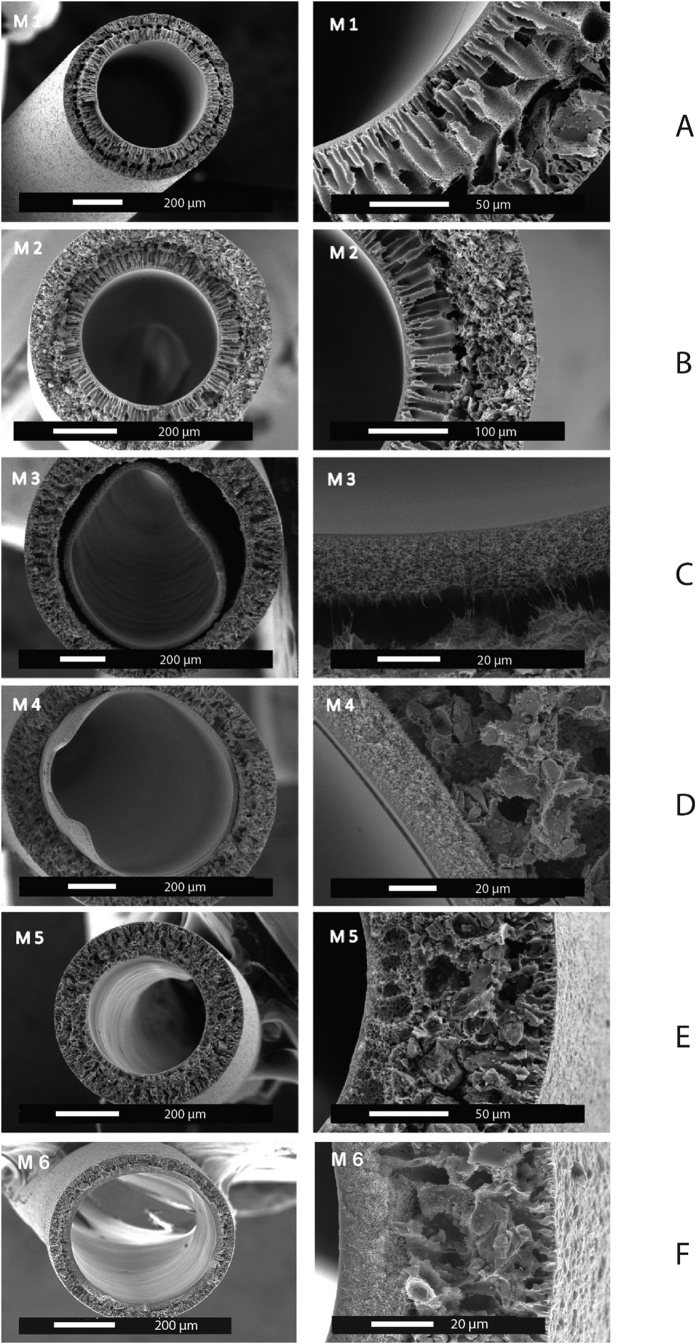
Scanning electron microscopy images of double layer mixed matrix membranes (see Table 1 for details of fabrication).

Six batches of the dual layer hollow fiber membranes were produced by a dry-wet spinning method. The spinning conditions were tuned to obtain membranes featuring optimal particle loading and morphological characteristics. As the adsorbent material Norit A Supra was used as it has high adsorption capacity and selectivity to creatinine and PBUT^19^,^20^. Table 1 summarizes the conditions under which membranes were fabricated and Fig. 1 presents typical SEM images of the resulting hollow fibers.

The first membranes (M1) produced using the new spinneret (see Fig. 1A) had low activated carbon (AC) loading of the outer membrane layer. Additionally, the ratio between the outer and inner membrane layers was not optimal. Double layer hollow fibers should have a thin inner layer to minimize mass transfer resistance and a thick outer mixed matrix to improve adsorption capacity. Consequently, the adaptation of the inner and outer layer pumping speeds resulted in a thinner inner layer and increased the thickness of MMM layer. Moreover, the loading of the AC was gradually increased from 37.5 to 60% in the outer membrane layer (see membrane M2, Fig. 1B). A higher AC content of the outer layer should improve the overall adsorption capacity of the hollow fibers. It was noticed that an activated carbon loading of higher than 60% sharply increases the brittleness of the membranes. The PVP content of the polymer dopes was also increased from 5 to 7% to avoid formation of undesirable macro-voids in the inner membrane layer.

M3 membranes, which were the next step in MMM optimization, were prepared using increased pumping speed for the outer layer. That resulted in noticeable separation of the inner and outer layers, so the polymer pumping speed was decreased back to 1.6 ml/min for the subsequent membrane batches (M4, M5, M6), while keeping the ratio 1:4 of the inner to outer layer pumping speeds (Fig. 1D). The adhesion of the inner and outer layers of the M4 membranes was better than for M3. However, delamination could still occur due to significant difference in viscosities of polymer dopes. Therefore, it was decided to decrease the PVP content of the inner layer back to 7%.

For the M5 membrane, there was no noticeable interface between the inner and outer layers (Fig. 1E). In other words, by decreasing the viscosity difference between the two extruded polymer solutions, we were able to avoid skin formation between the two membranes. In fact, the M5 membranes have a more interconnected structure than all previous membrane batches. Moreover, the introduction of the collecting wheel during the spinning and the reduction in the bore liquid pumping speed resulted in noticeably smaller diameter in comparison to M4 (376 μm vs 759 μm, respectively). The M5 membranes, similar to M3 and M4, were prepared with bore liquid composition that consists of 60% NMP and 5% PVP in pure water. Due to the presence of the solvent NMP in the bore liquid, demixing of the polymer is delayed resulting in an inner layer with a sponge-like structure and higher transport. Unfortunately, the ultrafiltration coefficient of the membranes was too high, reaching values up to 183 ml/h/m^2^/mmHg (M5) with noticeable loss of plasma proteins during filtration of human plasma through the membrane. The flux of the membrane could become lower when using water as the bore liquid and the parameters that are described in Table 1. The M6 membrane has the optimal characteristics required for our application: low ultrafiltration coefficient, no albumin leakage and small diameter and therefore will be used in further studies of solute transport (see Table 2 for a comparison of the M6 to the earlier MMM^20^ and to the low flux dialysis membranes F8HPS used currently in clinical practice). In fact, M6 has a low ultrafiltration coefficient in comparison to Fresenius F8HPS and previously reported membranes, and can be classified as low-flux membrane with MWCO around 12 kDa (see Fig. 1 of the appendix).

### Creatinine removal by the membranes

**Figure 2:**
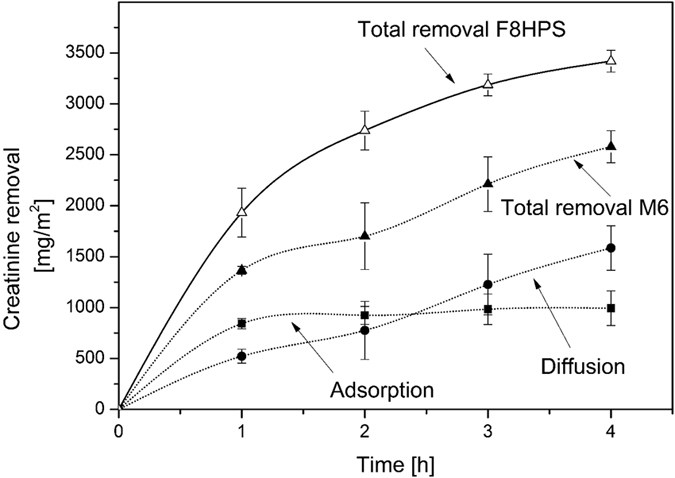
Comparison of the creatinine removal of mixed matrix membrane (dotted line) vs industrial membrane (solid line). The error bars indicate standard deviation of single experiment using multiple modules (n = 4).

For the M6 membrane, creatinine is removed by the combination of diffusion and adsorption (see Fig. 2) consistent to the results of earlier study^20^. After four hours, the M6 membranes remove 2549 mg/m^2^ of creatinine in comparison to the 3420 mg/m^2^ removed by the F8HPS membrane. This difference is due to the difference between the ultrafiltration coefficients of the two membranes: 3.4 ml/h/m^2^/mmHg for the M6 membranes vs 10.0 ml/h/m^2^/mmHg for the F8HPS. Clearly, the more open the membrane structure the higher the transport rate of the small solute, like creatinine, through the membrane. However, it is important to note here that although the F8HPS membrane has three times the ultrafiltration coefficient of MMM, it only removes 30% more creatinine in four hours of treatment, while in the first hour of the experiment, during which M6 membranes have maximum adsorption, there is small difference in creatinine removal between two membranes. In other words, if the adsorption capacity of the AC will be bigger, for example by the use of smaller particles with higher surface area, difference in creatinine removal between the two types of membranes is expected to be smaller than 30% percent. We believe that this result indicates the strong additive effect of the adsorption to the removal, which becomes even more remarkable for the removal of the protein-bound toxins (see next section).

Using the first generation of MMM^20^, creatinine removal was 2825 mg/m^2^, only 9% higher than the M6 membrane, even though these membranes had 23 times the ultrafiltration coefficient. We think that the excellent performance of the M6 membranes is attributed to small inner wall thickness. The selective inner layer is much thinner in comparison to previous generation of the fibers (Table 2) and so is the diffusion length of the solutes to the activated carbon, which is responsible for the adsorptive removal.

### Protein-bound toxin removal by the membranes

**Figure 3:**
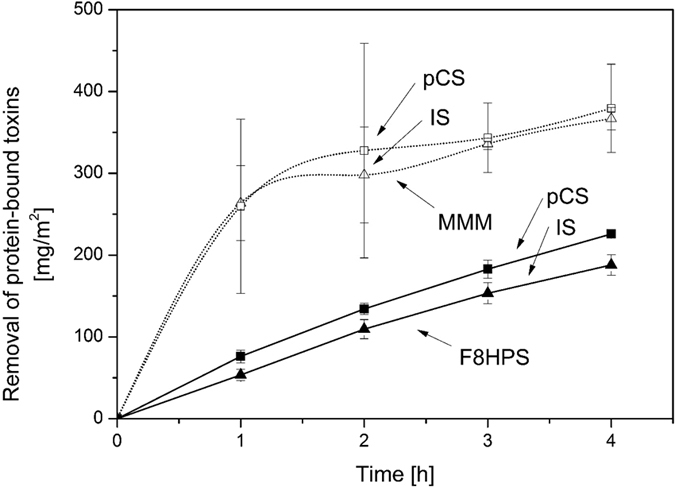
Removal of protein-bound toxins by F8HPS and mixed matrix membrane over time . Error bars indicate standard deviation of one experiments using multiple modules (n = 4).

In the case of the water-soluble creatinine, the low ultrafiltration coefficient of the MMM results in a lower rates of removal. However, in the case of the PBUT the pore size of the membranes and their ultrafiltration coefficient should have little influence on the removal of substances bound to albumin^21^,^22^,^23^. Figure 3 compares the removal of a mixture of IS and pCS from human plasma by the M6 and the F8HPS membrane. The M6 membranes have a very high rate of removal of 367 mg/m^2^ for IS and 380 mg/m^2^ for pCS in comparison to the Fresenius dialysis fiber (removal of 187 mg/m^2^ and 225 mg/m^2^ of IS and pCS respectively) and the previous generation of the mixed matrix membranes^20^ (252 mg/m^2^ and 160 mg/m^2^ of IS and pCS respectively). For our M6 membranes, the removal is entirely due to adsorption since in four hours of the experiment we did not detect any IS and pCS in the dialysate compartment. For the F8HPS membranes, the removal is due to diffusion of the toxins to the dialysate compartment. For the M6 membrane, the PBUT removal is the highest during the first hour of the experiment, but similarly to the creatinine removal, the activated charcoal saturation seems to occur in time. In case of the F8HPS membranes, the removal has constant rate throughout the four hours of the dialysis experiment.

The remarkably higher removal rates achieved by our membranes are due to the adsorption layer. In fact, during the experiment, the free fraction of the IS and pCS passes the inner selective membrane layer and is quickly adsorbed by the particles inside the outer layer. This keeps the concentration gradient for the removal very high and stimulates the release of the free fraction of toxins from the protein to the plasma, resulting in even higher removal. For F8HPS membranes, the removal of the free fraction of toxins is driven by diffusion, which also stimulates the release of the free fraction, but the concentration gradient is much lower. Meyer *et al*.^18^ showed that adding sorbent in the dialysate can also improve significantly the removal of PBUTs by the dialysis membrane. In fact, they predicted from modelling studies that adsorption could increase the driving force for the removal, leading to higher removal rates consistent with our findings reported here. However, as also highlighted by Meyer *et al*.^18^ the adsorbent particles needed to be well mixed to be able to adsorb the toxins that permeate through the membranes, especially at low concentrations. In the case of our MMM, the adsorption particles are evenly dispersed inside the membrane, thereby minimizing the diffusion length for the toxins to be removed and making the sorbent evenly available over the entire membrane surface. All our results strongly indicate the significant improvement of our membrane in comparison to first generation, and its great potential for the removal of PBUT.

**Figure 4:**
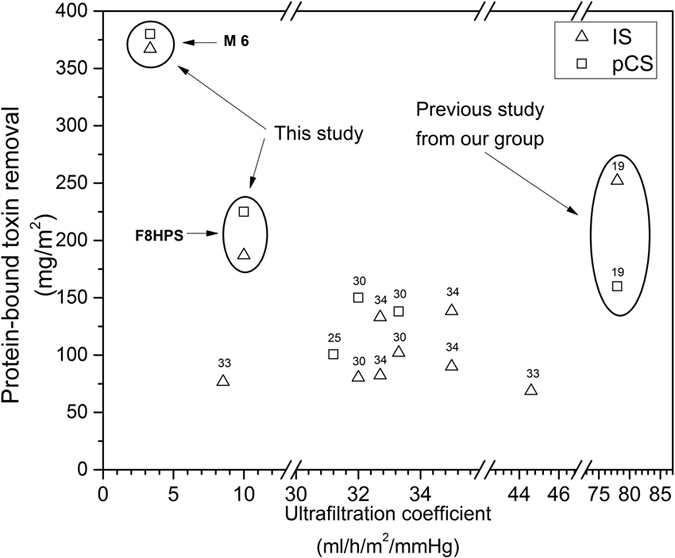
Removal of protein-bound toxins as a function of membrane permeability taken from multiple studies^20^,^26^,^30^,^31^,^32^. Ultrafiltration coefficients represent the type of membrane used in the studies. Some studies^30^ used different membranes to evaluate the influence of ultrafiltration coefficient on the PBUT removal. Others^32^ used same membranes under the varying conditions, such as 4 and 8 hours of dialysis treatment.

Figure 4 compares the removal of IS and pCS by the M6 membranes with that cited in various published papers, where researchers tried to improve the removal of PBUTs by using more open membranes, that had higher reduction ratios of the urea and creatinine^21^,^22^. The optimised M6 membranes of this study, as well as the first generation of MMM, have the highest removal for both toxins^20^. Figure 4 also shows clearly that the ultrafiltration coefficient of the standard dialysis membrane has no major effect on the removal of the PBUTs. It seems that only a small fraction of un-bound toxins is eliminated by diffusion through the membrane, so, contrary to small water-soluble molecules, the larger pore size seems to have little or no influence on the diffusive removal of PBUTs, due to the low concentration of the free fraction in the plasma. It is possible to augment their removal by prolonged dialysis treatment^24^,^25^, larger membrane surface area^23^,^26^ or/and by facilitating the diffusion of the toxins through the membrane^18^,^27^,^28^,^29^. Our mixed matrix membrane actually achieves the latter. It combines the enhanced mass transfer characteristics of the inner thin membrane layer (see Table 2) with the increased driving force for removal due to the adsorption in the outer layer, leading to superior performance in comparison to current state-of-the-art dialysis membranes.

## Conclusions and Outlook

This study presents the development of a new generation of mixed matrix hollow fiber membranes that can provide significant benefits to hemodialysis therapies in terms of the protein-bound toxin removal. These membranes offer superior performance in comparison to existing commercial dialysers during *in vitro* studies of four hours. In future, the new MMM will be assembled into the modules with high surface area and their performance will be investigated *in vitro* with full blood and *in vivo* using uremic goats.

## Methods

### Materials

For the preparation of the hollow fiber membranes, Ultrason E6020 polyethersulfone (PES, BASF, Ludwigshafen, Germany) and polyvinylpyrrolidone K90 (PVP, Sigma-Aldrich Chemie GmbH, Munchen, Germany) were dissolved in extra pure N-methylpyrrolidone (NMP, Acros Organics, Geel, Belgium). Activated carbon (Norit A Supra, Norit Netherlands BV, Amersfoort, the Netherlands) was sieved through 45 μm sieve (VWR, Amsterdam, the Netherlands) and used as an adsorbent in outer polymer layer. All polymer solutions were allowed to degas for at least 24 hours prior to membrane fabrication. Creatinine, indoxyl sulfate, human serum albumin (HAS), inulin, vitamin B12, α-lactalbumin and α-chymotrypsin were purchased from Sigma Aldrich. p-Cresyl sulfate was synthesized by the Laboratory for Organic and Bio-organic Synthesis at the Ghent University (Belgium) following the method described by Feigenbaum *et al*.^33^.

Human plasma from healthy vol unteer donors was obtained from Sanquin (Amsterdam, the Netherlands) in compliance with local ethical guidelines. To prepare the dialysate solution 2 mM KCl, 140 mM NaCl, 1.5 mM CaCl_2_, 0.25 mM MgCl_2_, 35 mM NaHCO_3_ and 5.5 mM glucose (all Sigma-Aldrich) were dissolved in ultra-pure water. Fresenius F8HPS low-flux dialysis membranes (kindly provided by FMC, Vlijmen, the Netherlands) were used as reference.

### Hollow fiber membrane preparation

**Figure 5:**
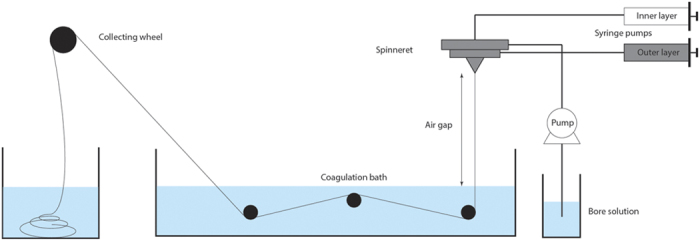
Schematic of the hollow fiber spinning set-up.

The dual layer hollow fiber membranes were produced by dry-wet spinning (Fig. 5). Two polymer solutions (one particle-free and one with activated carbon) were transferred into stainless steel syringes and left to degas overnight. The following day the syringes were mounted in the high-pressure syringe pumps and connected to a specially designed spinneret together with the bore solution. Subsequently, the spinneret was placed above the coagulation bath at a fixed height (air gap). The collection of the resulting hollow fibers was by the collecting wheel.

To improve surface-to-volume ratio of the membrane modules, the new generation of the MMM hollow fibers was produced via a newly developed spinneret, which ensured smaller dimension of the produced hollow fibers. Table 3 compares the characteristics of the new spinneret to that used in an earlier study^20^.

### Membrane module preparation

All fabricated membranes were washed in water to remove the remaining solvent and kept in ultra-pure water. Before module preparation, the membranes were dried in air at room temperature. Membrane modules were made by potting hollow fiber membranes inside the 14 cm long tubes with 2 Kartell T-connectors (VWR, Amsterdam, the Netherlands) located 2 cm from each end. As a result, the membrane modules had an effective length of 10 cm. Modules of MMM contained three hollow fibers (4.24 cm^2^) and modules of F8HPS ten hollow fibers (6.28 cm^2^). Each end was glued by water-clear polyurethane casting resin (Easy Composites Ltd, London, UK) and cut open after the glue had hardened.

### Membrane characterization

#### Ultrafiltration coefficient

Modules containing three MMM hollow fibers were pre-compacted with ultra-pure water at a trans-membrane pressure (TMP) of 1500 mmHg for at least one hour before measurements. After this, the amount of permeated water was measured over time under 375, 750, 1125 and 1500 mmHg of transmembrane pressure (TMP). The ultrafiltration coefficient for the resulting membranes was calculated as the slope of the linear fit of the flux (ml/m^2^/h) versus TMP graph.

#### Scanning Electron Microscopy

The morphological characteristics of the hollow fiber membranes were visualized by use of a scanning electron microscopy (SEM). The membranes were dried in air followed by fracturing in liquid nitrogen to reveal the cross-section. Subsequently, the samples were sputtered with gold using the Cressington 108 auto sputter (Cressington Scientific Instruments, Watford, UK) and examined using a Philips XL-30 ESEM-FEG Scanning Electron Microscope (Philips, Amsterdam, the Netherlands).

#### Cross-flow transport experiments

**Figure 6:**
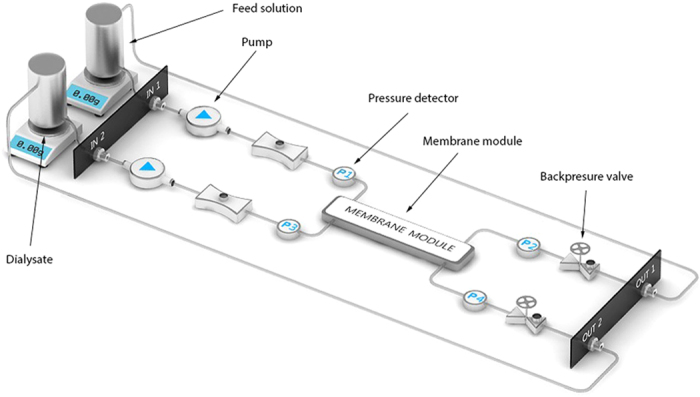
Schematic of the experimental set-up (drawing by Convergence).

Figure 6 presents the set-up used for the cross-flow transport experiments (Convergence, Netherlands). Experimental set-up consists of two peristaltic pumps, four pressure detectors and two back-pressure valves. All the parts of the set-up are connected via PTFE tubings. Feed and dialysate solutions are pumped to membrane module in counter-current mode of operation and their speeds are controlled by peristaltic pumps. Constant values of TMP are generated by the back pressure valves. TMP of the system is calculated following the formula below.


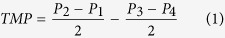


where P_1_ and P_2_ are the pressure values before and after membrane module for feed solution and P_4_ and P_3_ are corresponding values for dialysate.

Before the start of each experiment, the transport of clean water through the modules was measured for at least one hour under 750 mmHg of TMP to check whether all membrane bores were open. For the large diameter MMM it was really easy to see even with a light microscope whether all the bores are open. However, for the smaller Fresenius F8HPS fibers the light microscopy was not able to provide reliable results. For this reason, we performed clean water transport studies of modules with multiple fibers and we compared it to that with a single fiber. Increase in the number of fibers is expected to result in proportional increase of the flow values. Only modules with flow values proportional to number of fibers were considered open and were used in the experiments.

For the creatinine cross-flow measurements, the feed compartment contained 50 ml of 0.1 g/l creatinine solution in PBS while the dialysate compartment contained 50 ml PBS. Flow rates were set at 5 ml/min and 10 ml/min for feed and dialysate compartments, respectively. The TMP was kept at 0 mmHg by adjusting the backpressure valve.

Subsequently, 50 ml of human plasma, spiked with uremic concentrations (40 mg/l) of IS and pCS^34^, was applied at the feed side and protein-bound toxin removal was determined. Pre-prepared dialysate solution was used at the dialysate side of the set-up. Due to the viscosity of human plasma as compared to water, the flow rate of the feed solution was decreased to 1 ml/min to prevent pressure drops across the module.

Total creatinine and PBUT removal was calculated as the sum of diffusive and adsorptive removal. Diffusive removal was calculated by the concentrations of the solutes found in the dialysate, whereas the adsorptive removal was calculated as the difference between the total removal of the solutes from the “blood” side and the diffusive removal. It should be noted that for PBUTs total removal was equal to adsorptive removal since PBUTs were not detected in the dialysate side of the module. As the final step, all values of the PBUT and creatinine removal were normalized by the surface area as it has a direct influence on the creatinine and PBUT clearances^23^.

#### Quantification of creatinine and protein-bound uremic toxins

Creatinine (MW 113) concentrations were analysed by standard laboratory methods using the UV detection at 254 nm.

The concentrations of IS (MW 212 Da, protein bound ~97%^13^) and pCS (MW 187 Da, protein bound ~95%^13^) were determined as described by Meert *et al*.^27^. In short, plasma samples were deproteinized by heat treatment, filtered through 30 kDa filters (Amicon Ultracel-30 K, Merck Millipore Ltd) and subsequently analysed by reverse-phase high-performance liquid chromatography (RP-HPLC). Concentrations were measured by fluorescence analysis (IS: λ_ex_ = 280 nm, λ_em_ = 340 nm; pCS: λ_ex_ = 265 nm, λ_em_ = 290 nm).

#### Molecular weight cut-off (MWCO) of the membranes

The molecular weight cut-off of the membranes was estimated by filtration of marker molecules of various sizes: HSA, α-chymotrypsin, α-lactalbumin, inulin and vitamin B12. A series of the solutions was prepared by slow dissolution of the powdered molecule in the PBS solution. Concentrations of the solutions were determined both before and after the ultrafiltration experiments by the use of the UV-vis. More information can be found in the Table 4.

Ultrafiltration experiments were performed as follows. MMMs were pressurized by demi-water under 1500 mmHg for two hours prior to the experiment. As a next step, a solution of each of the marker molecules was filtered through the MMM in a dead-end setting at 375 mmHg. After 1 ml of the solution had permeated through the membrane, sieving coefficients were calculated as:


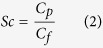


swhere C_p_ and C_f_ are the protein concentrations in the permeate and feed solutions respectively.

### Statistics

Results are presented as average values of single experiment using multiple modules (n = 4) and their corresponding standard deviations. Comparative statistical analysis was performed using the IBM SPSS Statistics 22 software package. Statistical difference was determined by independent samples t-test, with the significance level set to P < 0.05.

## Additional Information

**How to cite this article**: Pavlenko, D. *et al*. New low-flux mixed matrix membranes that offer superior removal of protein-bound toxins from human plasma. *Sci. Rep.* **6**, 34429; doi: 10.1038/srep34429 (2016).

## Supplementary Material

Supplementary Information

## Figures and Tables

**Table 1:** Spinning conditions of the various double layer membranes.

	M 1	M 2	M 3	M 4	M 5	M 6
Inner layer (PES/PVP)	15/5	15/7	15/10	15/10	15/7	15/7
Outer layer (PES/PVP/AC)**	14/1.4/37.5	14/1.4/60	14/1.4/60	14/1.4/60	14/1.4/60	14/1.4/60
Inner layer pumping speed (ml/min)	0.5	0.4	0.4	0.4	0.4	0.4
Outer layer pumping speed (ml/min)	1	1.6	2	1.6	1.6	1.6
Bore liquid pumping speed (ml/min)	1.2	1.2	2	2	1.2	1.2
Bore composition*	B1	B1	B2	B2	B2	B1
Air gap (cm)	15	15	10	10	10	10
Spinneret type	Spinneret 2.0	Spinneret 2.0	Spinneret 2.0	Spinneret 2.0	Spinneret 2.0	Spinneret 2.0
Collecting speed (m/min)	Free fall	Free fall	Free fall	Free fall	3.5	3.5

^*^Bore liquid B1 consists of pure water and the bore liquid B2 contains 60% NMP and 5% PVP in pure water.

^**^Amount of activated carbon was calculated in relation to the amount of PES.

**Table 2:** Properties of the mixed matrix and industrial membranes.

	Mixed matrix membranes (M6)	F8HPS	Tijink *et al*.^1^
Lumen diameter (μm)	450	200	669
Inner layer (μm)	21	40	49
Outer layer (μm)	47	N/A	111
Ultr. coeff. (ml/h/m^2^/mmHg)	3.35	10	78
Albumin leakage	NO	NO	YES
Creatinine removal after 4 hours (mg/m^2^)	2579	3420	2825

**Table 3:** Comparison of the spinneret specifications.

	Spinneret 1^20^	Spinneret 2 (*this study*)
Inner needle diameter (mm)	0.26	0.16
Outer needle diameter (mm)	0.46	0.26
Inner diameter first orifice (mm)	0.66	0.46
Outer diameter first orifice (mm)	0.96	0.66
Inner diameter second orifice (mm)	1.66	0.86

**Table 4:** Experimental conditions for the hollow fiber MWCO determination.

Marker molecule	MW (kDa)	Concentration (mg/ml)	UV wavelength (nm)
HSA	66.5	1	280
α -chymotrypsin	25	0.1	280
α -lactalbumin	14.2	1	280
Inulin	5	0.1	285
B12	1.3	0.1	550
