# Supplementary material for: New low-flux mixed matrix membranes that offer superior removal of protein-bound toxins from human plasma
Source: Sci Rep. 2016 Oct 5;6:34429. doi: 10.1038/srep34429 (PMC5050520; doi:10.1038/srep34429)
Supplement: Supplementary Information [file srep34429-s1.docx]

New low-flux mixed matrix membranes that offer superior removal of protein-bound toxins from human plasma

Denys Pavlenko^1^, Esmée van Geffen^1,2^, Mies J. van Steenbergen^3^, Griet Glorieux^4^, Raymond Vanholder^4^, Karin G.F. Gerritsen^2^, Dimitrios Stamatialis^1^

^1^Department of Biomaterials Science and Technology, MIRA Institute for Biomedical Engineering and Technical Medicine, University of Twente, P.O. Box 217, 7500 AE Enschede, The Netherlands

^2^Department of Nephrology and Hypertension, University Medical Centre Utrecht, P.O. Box 85500, 3508 GA Utrecht, The Netherlands

^3^Department of Pharmaceutics, Utrecht Institute for Pharmaceutical Sciences, Utrecht University, P.O. Box 80082, 3508 TB Utrecht, The Netherlands

^4^Ghent University Hospital, Department of Internal Medicine, Nephrology Division, 9000 Ghent, Belgium

**Appendix**

The SC curve of the M6 mixed matrix membranes. The MWCO of the membranes is around 12 kDa

sdf

**
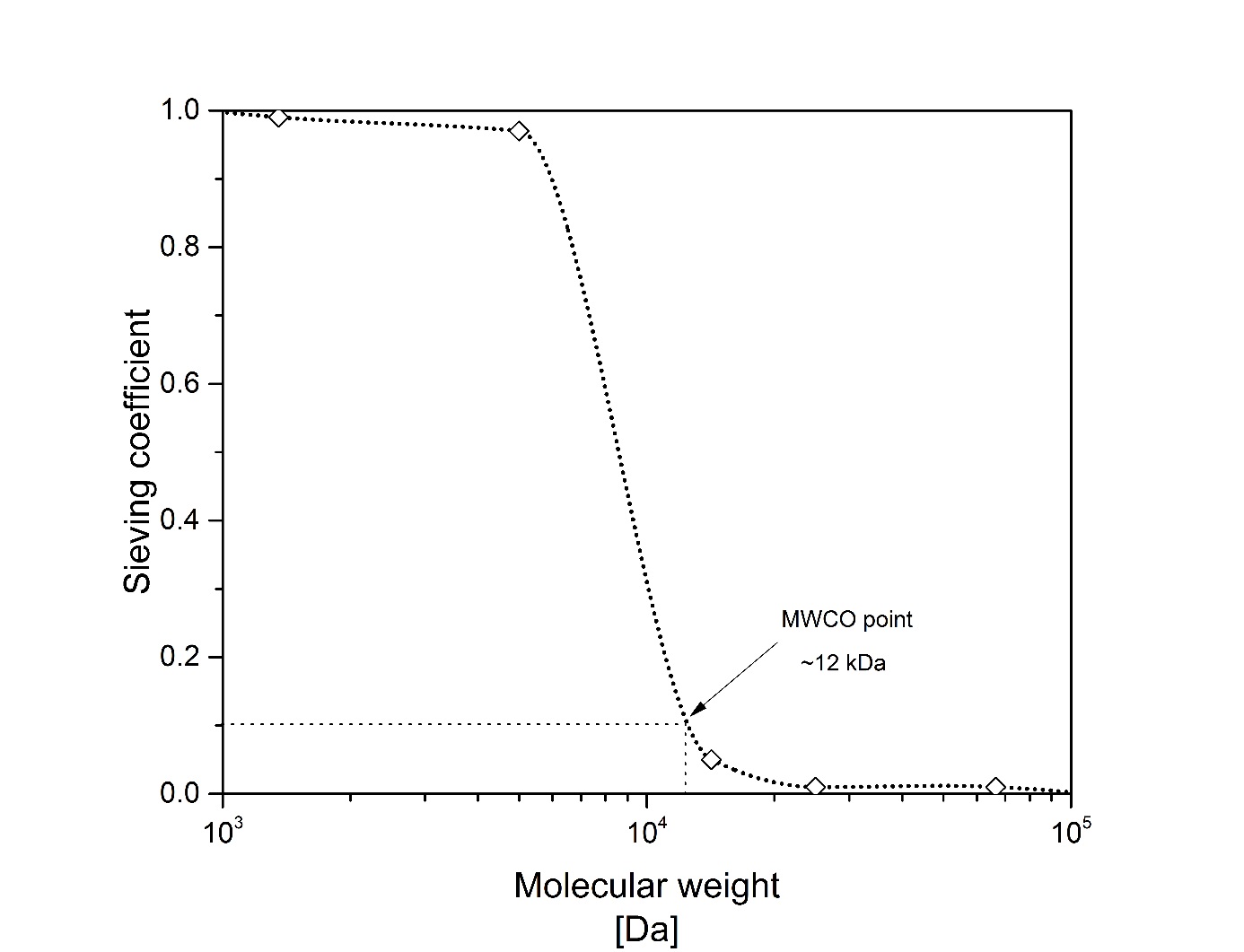
**

Figure 1. Sieving curve for the M6 MMM membranes.
